# Recovery of performance and persistent symptoms in athletes after COVID-19

**DOI:** 10.1371/journal.pone.0277984

**Published:** 2022-12-07

**Authors:** Shirin Vollrath, Daniel Alexander Bizjak, Jule Zorn, Lynn Matits, Achim Jerg, Moritz Munk, Sebastian Viktor Waldemar Schulz, Johannes Kirsten, Jana Schellenberg, Jürgen Michael Steinacker

**Affiliations:** 1 Division of Sports and Rehabilitation Medicine, Department of Medicine, Ulm University Hospital, Ulm, Germany; 2 Clinical & Biological Psychology, Institute of Psychology and Education, Ulm University, Ulm, Germany; Universita degli Studi di Milano, ITALY

## Abstract

**Introduction:**

After the acute Sars-CoV-2-infection, some athletes suffer from persistent, performance-impairing symptoms, although the course of the disease is often mild to moderate. The relation between cardiopulmonary performance and persistent symptoms after the acute period is still unclear. In addition, information about the development of this relationship is lacking.

**Objective:**

To assess the prevalence of persistent symptoms over time and their association with the performance capability of athletes.

**Methods:**

We conducted two cardiopulmonary exercise tests (CPET) in a three months interval with 60 athletes (age: 35.2±12.1 years, 56.7% male) after infection with Sars-CoV-2 (t_0_: study inclusion; t_1_: three months post t_0_). At each examination, athletes were asked about their persistent symptoms. To evaluate the change of Peak VO_2_/BM (Body Mass) between the time before infection and the first examination, the VO_2_/BM (predVO_2_) before infection was predicted based on anthropometric data and exercise history of the athletes. For data analysis, athletes were grouped according to their symptom status (symptom-free, SF; persistent symptoms, PS) and its progression from the first to the second examination 1) SF-SF, 2) PS-SF and 3) PS-PS.

**Results:**

Comparing the SF and PS groups at t_0_, significant differences for Max Power/BM, Max Power/lbm (lean body mass), Peak VO_2_, Peak VO_2_/BM, Peak VO_2_/lbm, Peak VO_2_/HR, Peak VE, Peak Vt and VE/VCO_2_-Slope were observed. Regarding the progression over three months, an increase in Max Power/BM was shown in SF-SF and PS-SF (tendency). Max Power/lbm increased in SF-SF and PS-PS (tendency). A decrease of VE/VCO_2_-Slope in PS-PS was found.

**Conclusion:**

COVID-19 led to a decline in performance that was greater in PS than in SF. Additionally, PS had decreased ventilatory parameters compared to SF. Furthermore, an improvement over time was observed in some CPET parameters and a partial recovery was observed judging by the decrease in various symptoms.

## Introduction

The long-term sequelae of COVID-19 are manifold and patients suffer from the symptoms for up to 12 weeks after infection (Long-COVID) or even longer (Post-COVID) [1–3]. Not only people who were hospitalized in the acute period, even people with a mild or moderate disease course can suffer from Long-COVID [4]. Furthermore, athletes, who mostly do not have comorbidities, can be seriously affected by COVID-19 [5, 6]. In addition, symptoms can also occur for the first time after recovery from the infection [1], resulting in a lower performance capability of patients and athletes [7–10].

The limitations reported by patients vary in severity and symptomatic expression [11, 12]. To exclude organic restrictions and / or to evaluate the performance capability of the cardiopulmonary and respiratory system, cardiopulmonary exercise testing (CPET) can be conducted [13]. Previous research focused on various CPET variables like breathing reserve, Respiratory Exchange Ratio, Peak VO_2_, Peak Heart Rate or PETCO_2_ [14–16]. CPET is already recommended and used in Long-COVID and Post-COVID studies to assess the limitations in the cardiopulmonary and respiratory system after COVID-19 [17–21]. To gain insights how persistent symptoms develop over time, it is important to monitor patients who have previously been infected with Sars-CoV-2 over at least several months up to several years by measuring objective performance parameters, like Peak VO_2_ or VE/VCO_2_-Slope over time [22].

Although the majority of athletes represent a healthy part of the general population, they have an increased need for health monitoring because they expose their bodies to increased loads during heavy exercise, training and competition. However, there is only limited knowledge about the development of the performance and cardiopulmonary function of athletes after a Sars-CoV-2 infection, especially when athletes suffer from persistent symptoms.

Therefore, this study aims to evaluate the athletes’ symptom state, cardiopulmonary function, and performance capacity after infection and three months later. Thus, the predicted performance capacity before infection was compared with the performance capacity post-infection. Furthermore, it was of interest whether athletes with persistent symptoms have decreased cardiopulmonary function and performance. In addition, the relationship between the predicted aerobic capacity and decreased infection-related performance was analyzed. Finally, the development of the recovery process of athletes with and without persistent symptoms over three months was studied.

## Material and methods

The study was conducted at the Division for Sports and Rehabilitation Medicine, Center of Internal Medicine of the University Hospital in Ulm, Germany. All athletes were participants of the CoSmo-S study (COVID-19 in German Competitive Sports) [23]. The inclusion criteria were 1) Age ≥ 18 years, 2) Sport at least three times per week (20 metabolic equivalents (METs) / week), 3) confirmed Sars-CoV-2 infection but at least > 2 weeks after a positive PCR-test. Further details of the inclusion / exclusion criteria and the study design can be found in the study protocol by Niess et al. [23].

### Ethical approval

All participating athletes took part voluntarily and gave informed consent prior to inclusion. The study was performed in accordance with the Declaration of Helsinki. The study was approved by the ethics committee of Ulm University (EK 408/20).

### Investigation period

The period of investigation was between June 2020 and January 2022, but the examination of study participants is still ongoing at the time of submission of this manuscript. All athletes who had at least two examinations, in a three months interval, with CPET until January 2022 were included in this pilot evaluation.

### Study population

In total, 60 persons were included (56.7% male). There were two time points of investigation: The first one (t_0_) (4.1 ± 3.8 months after infection) was at the day of study inclusion; the second one (t_1_) three months later (3.3 ± 0.5 months).

### Examination of symptoms

At both examination dates, the athletes were asked about the presence of persistent symptoms based on the international consensus criteria for myalgic encephalomyelitis / chronic fatigue syndrome and medical history evaluation [24]. The symptoms were differentiated into eight symptom categories (*Fatigue and performance decrease*, *Sleeping disorders*, *Neurocognitive disorders*, *Respiratory disorders*, *Autonomic disorders*, *Pain*, *Psychological-related items*, *Immunological disorders)*. The severity of the symptoms is not rated in this questionnaire. All symptoms assigned to the different categories appeared for the first time after or during COVID-19. Once a symptom was mentioned, it was documented as "present" in the respective category and the participant was grouped into the category Persistent Symptoms (Group PS). Athletes who reported no symptoms related to COVID-19 at t_0_ were assigned to the symptom-free group (Group SF). The athletes could report multiple persistent symptoms. Thus, an athlete could be listed in several symptom categories.

### Examination of body composition

For measuring weight and body fat, a bio-impedance-scale (InBody 770, InBody Europe B.V., Eschborn, Germany) was used. Lean Body Mass (lbm) was calculated as follows: lbm (kg) = weight (kg)–body fat (kg). Lean body mass was used to diminish differences by gender.

### Prediction of peak oxygen consumption before infection

The VO_2_ Peak before infection (predVO_2_) was predicted by three different experts blinded to all information except the kind of sport, training and performance data before infection from an athletes-questionnaire, medical history and anthropometric data. They estimated the VO_2_ Peak (ml/min/kg/BM) in intervals with a width of 5 ml in the range from “≤ 15 ml” to “> 65 ml”.

### CPET

The physical performance was tested by a CPET, conducted with the breath-by-breath (Ergostik, Geratherm Respiratory, Bad Kissingen, Germany). All examinations were conducted on a cycling ergometer (Excalibur Sport, LODE B.V., Groningen, Netherlands). The ramp protocol was chosen according to the estimated fitness level, age, sex and weight of the athletes so that total exhaustion was reached in the desired time (8–12 min). The same protocol was chosen at the second measuring point. All CPETs were evaluated by the same examiner. To set individual reference values like calculated MVV (maximal voluntary volume) and IC (inspiratory capacity) for the athletes, a spirometry was conducted before CPET using the same device.

The following variables were measured: Max Power/BM (W/kg BM), Max Power/lbm (W/kg lean body mass), Peak VO_2_ (l/min), Peak VO_2_/BM (ml/min/kg BM), Peak VO_2_/lbm (ml/min/kg lean body mass), Peak Heart Rate (HR) (1/min), Peak VO_2_/HR (ml/beat), Peak Ventilation (VE) (l/min), Peak Tidal Volume (Vt) (l/breath), Peak Breathing frequency (Bf) (1/min), Peak Tidal Volume / Vital capacity (Vt/VC) (%), Ventilation/Volume of CO2 –Slope (VE/VCO_2_-Slope).

### Change of performance over three months

To determine whether the CPET variables changed over three months, three subgroups, depending on their symptom status were formed in terms of progression: At t_0_ persistent symptoms and at t_1_ symptom-free → PS-SF, both at t_0_ and t_1_ persistent symptoms → PS-PS, both at t_0_ and t_1_ symptom-free → SF-SF.

### Statistics

The statistical analysis was performed using IBM SPSS Statistics, version 28.0.0.0 (IBM Deutschland GmbH, Ehningen, Germany). Graphs were created with R version 4.1.1 (R Core Team, 2020).

To evaluate the correlation of the different experts who estimated the predVO_2_, a Spearman rho test was conducted. PredVO_2_ was calculated from the mean of the estimated intervals of each rater. To calculate the difference between predVO_2_ and Peak VO_2_/BM in SF and PS, a sign-test was conducted. To evaluate whether a difference in predVO_2_ between SF and PS exists a Mann-Whitney-U test was conducted.

To examine the differences in CPET parameters between the different symptom groups (PS: atheltes with persistent symptoms, SF: symptom-free athletes), t-tests and Mann-Whitney-U tests were calculated. To control for possible confounding variables (time since infection and age), robust linear regression models were conducted.

To evaluate whether there is a change of the CPET variables in each group over three months, a paired two-tailed t-test as well as a paired Wilcoxon-test were used.

Cohen’s d was calculated as effect size of the differences between the variables.

Missing values were always excluded in pairs in the analyses.

The significance level for all tests was set at (p<0.05).

## Results

### Progression of symptoms

**Fig 1:**
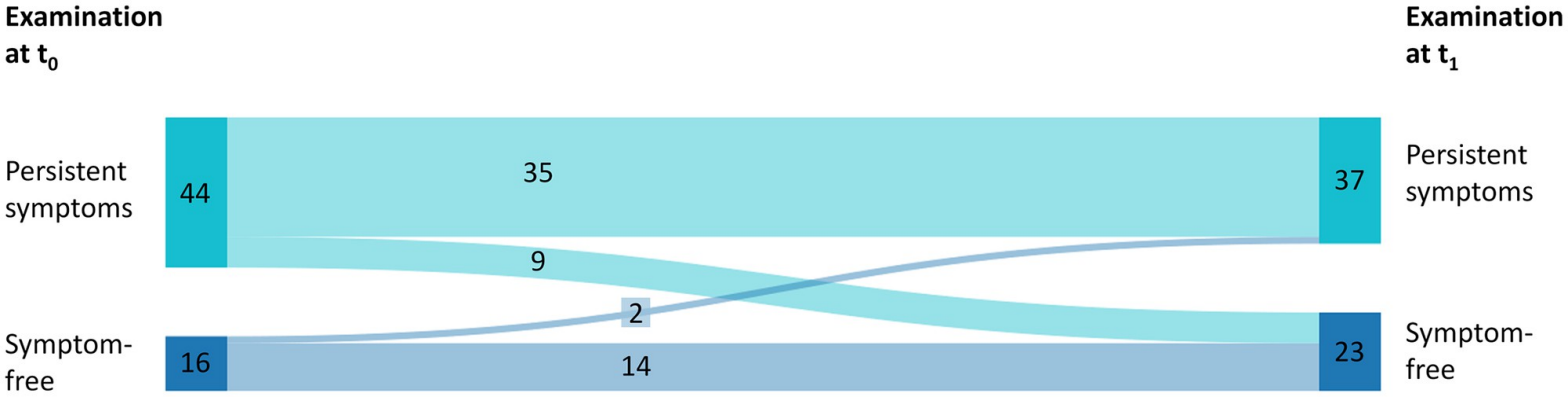
Development of symptom status. Symptom status of all 60 athletes at t_0_ (first examination date) and t_1_ (3.3 ± 0.5 months post first examination). 35 of 44 athletes, who had persistent symptoms at t_0_ still stated persistent symptoms at t_1_ (progression group PS-PS). Nine athletes became symptom-free over three months (progression group PS-SF). 14 of the 16 athletes remained symptom-free over the observation period (SF-SF). Two athletes developed symptoms over time. Data were collected with medical history and the International Consensus CFS questionnaire [24].

In total, 60 athletes were included. However, not all parameters could be used for the analysis due to implausible values during measurement or missing values. These values have been excluded from the analysis presented in this study. At the first examination (t_0_), 16 of the 60 athletes were symptom-free. At t_1_, 23 athletes were symptom-free, of which nine athletes previously had symptoms at t_0_. While 44 athletes reported suffering from at least one symptom at t_0_, this number decreased to 37 athletes at t_1_. Fig 1 shows the number of athletes with or without symptoms at the two examinations.

### Symptom categories

**Fig 2:**
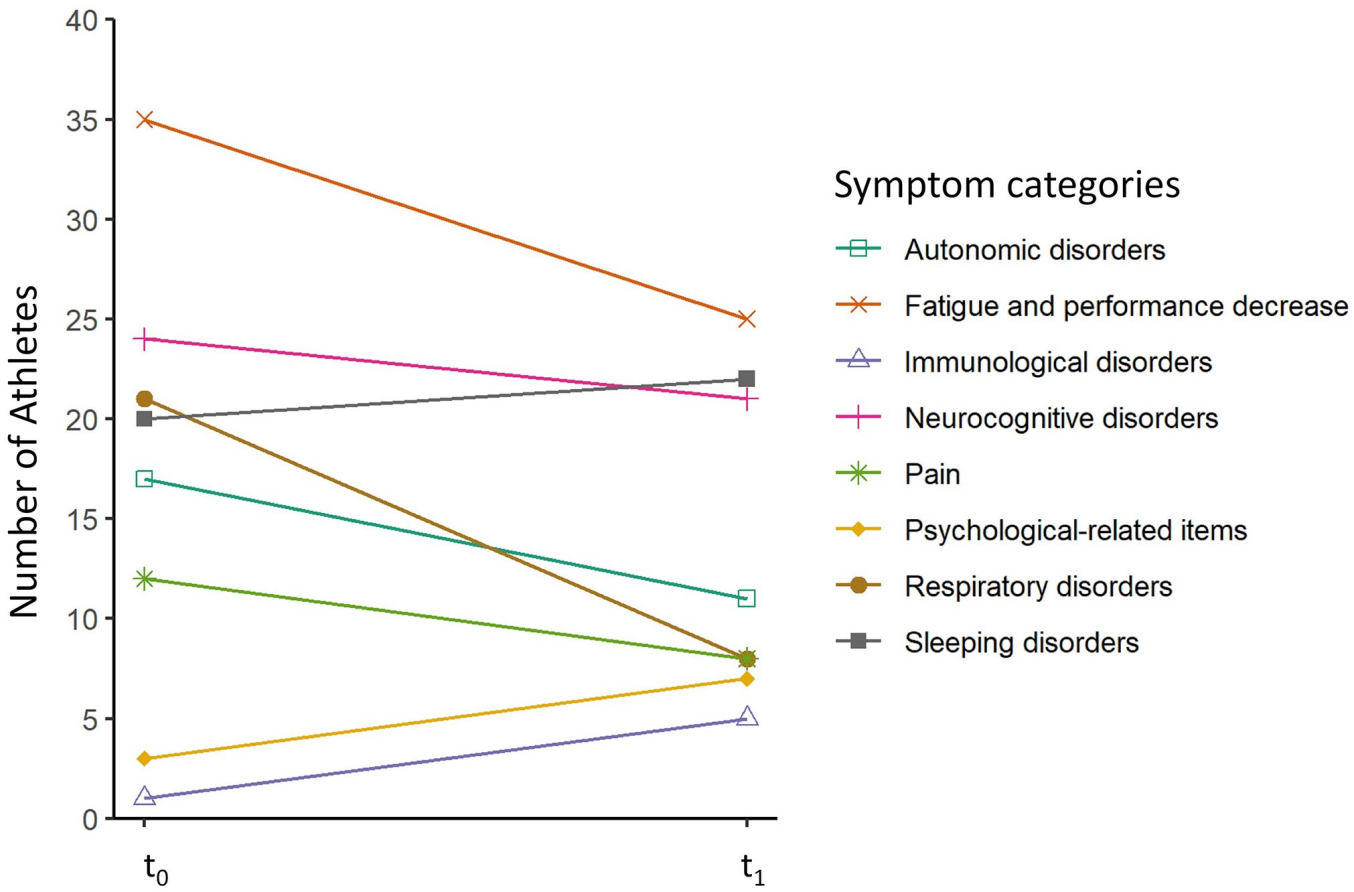
Course of symptom categories over investigation period. Number of athletes who stated at least one symptom in the eight symptom categories at t_0_ (first examination date) and t_1_ (3.3 ± 0.5 months post first examination). Data of symptom categories were collected with medical history and the International Consensus CFS questionnaire [24]. In total 44 athletes stated at least one symptom at t_0_ and 37 athletes at t_1_ (3.3 ± 0.5 months post first examination). Athletes could state symptoms in multiple categories.

Fig 2 shows the number of athletes at both measurement points who reported at least one symptom in the corresponding symptom category. With the medical history and the International Consensus CFS questionnaire [24], athletes were asked about persistent symptoms after COVID-19. It was possible to report symptoms for more than one category. The number of athletes with symptoms in the categories Fatigue and performance decrease, Neurocognitive disorders, Respiratory disorders, Autonomic disorders and Pain decreased over time. Contrary to that, the number of athletes with symptoms in the categories sleeping disorders, psychological-related items and immunological disorders increased over time.

### Number of symptom categories

Among the athletes who had persistent symptoms at t_0_, the highest number of athletes (n = 11) had symptoms that belonged to two symptom categories, closely followed by both four symptom categories (n = 10) and one symptom category (n = 10). At t_1_, 11 athletes had symptoms from one symptom category, and nine athletes had symptoms from three symptom categories. The highest numbers of symptom categories were seven at t_0_ and eight at t_1_.

### Prediction of peak oxygen consumption before infection

**Fig 3:**
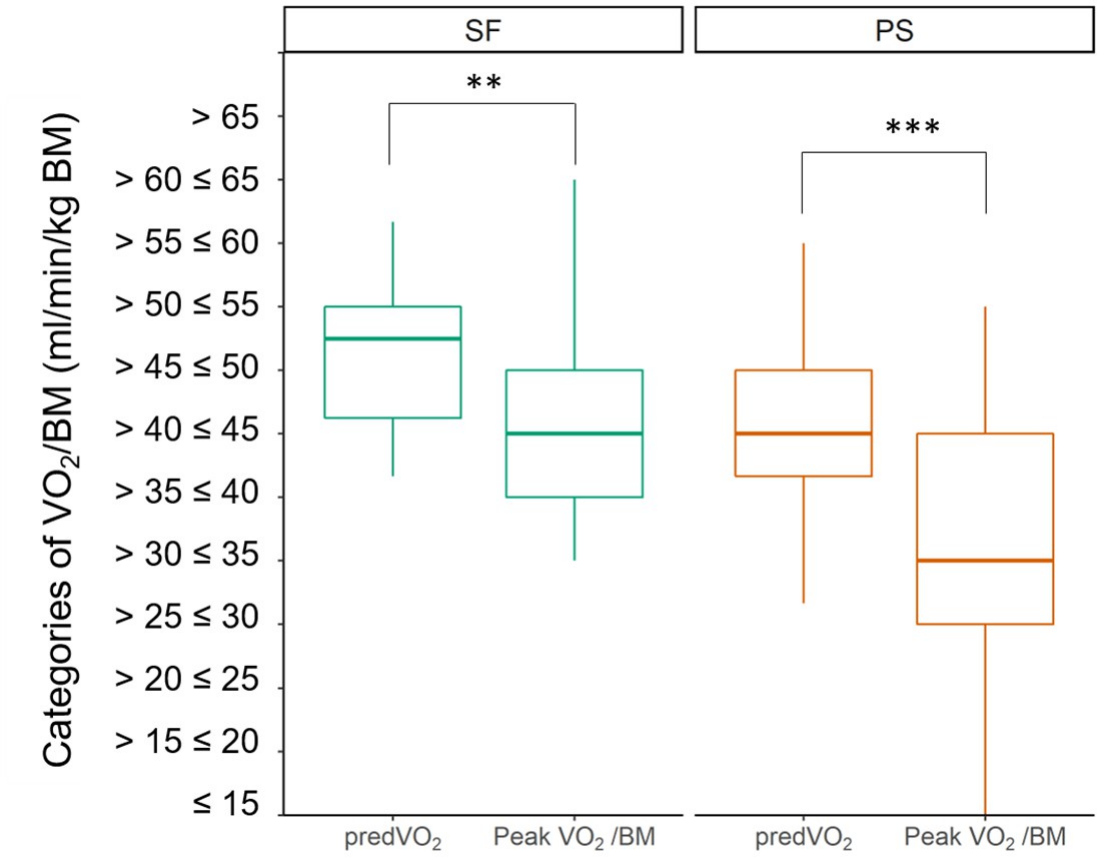
Differences between predVO_2_ and Peak VO_2_/BM. Significant differences of means between the calculated Peak VO_2_ (predVO_2_) and in PS and SF at t_0_ (first examination). In both groups the measured values were significantly below the values predicted for the time before infection. **p<0.01, ***p<0.001.

The results of predVO_2_ of the three different expert raters correlated significantly (p<0.001). The correlation factor shows how well the raters correspond with each other: r_sp_ = 0.800, r_sp_ = 0.628 r_sp_ = 0.537. Fig 3 shows the means of the intervals of predVO_2_ and Peak VO_2_/BM at t_0_ for symptom-free athletes and athletes with persistent symptoms. The prediction of predVO_2_ differed significantly between SF and PS (p = 0.015). In both groups, there were differences between predVO_2_ and Peak VO_2_/BM (SF: p = 0.004, PS: p<0.001). In SF, the means of predVO_2_ and Peak VO_2_/BM were in the intervals “> 45ml ≤ 50ml” and “> 40ml ≤ 45ml”, respectively. In PS, the means of predVO_2_ and Peak VO_2_/BM were in the intervals “> 40 ≤ 45ml” and “> 30ml ≤ 35ml”, respectively. In 45 athletes, Peak VO_2_/BM was decreased at least one interval (~5 ml/min/kg BM) compared to the value predicted for the time before the infection. Of these, 19 athletes had a peak VO_2_/BM lower by more than 10 ml/min/kg BM compared to predVO_2_, and two athletes had a deficit of more than 25 ml/min/kg BM compared to predVO_2_. Athletes who were symptom-free at t_0_ had a lower decrease of Peak VO_2_/BM than athletes with persistent symptoms. In eleven athletes, predVO_2_ was one interval lower or at the same interval as measured at t_0_.

### CPET

Table 1 shows the anthropometric and CPET data of all athletes at t_0_. The 35.15 (±12.14) years old population had a mean of 36.80ml (±10.53ml) maximal oxygen consumption and was able to perform 3.66W (±1.12W) per kilogram body mass.

**Table 1:** Anthropometric and CPET data of study population (N = 60) at t_0_ (first examination date). Values are given as mean and standard deviation (SD).

Anthropometrics & CPET variables	N	Mean (±SD)
**Age (years)**	60	35.15 (±12.14)
**Body Mass (kg)**	60	74.78 (±15.11)
**Height (cm)**	60	175.75 (±9.05)
**Body Mass Index (kg/m** ^**2**^ **)**	60	24.03 (±3.61)
**Lean Body Mass (kg)**	58	59.12 (±11.59)
**Max Power/BM (W/kg BM)**	60	3.66 (±1.12)
**Max Power/lbm (W/kg lbm)**	58	4.59 (±12.14)
**Peak VO**_**2**_ **(l/min)**	56	2.74 (±0.87)
**Peak VO**_**2**_ **/BM (ml/min/kg BM)**	56	36.80 (±10.53)
**Peak VO**_**2**_ **/lbm (ml/min/ kg lbm)**	55	46.04 (±9.77)
**Peak HR (1/min)**	52	171.69 (±14.87)
**Peak VO**_**2**_ **/HR (ml/beat)**	50	15.58 (±4.64)
**Peak VE (l/min)**	60	106.48 (±35.16)
**Peak Bf (1/min)**	60	39.77 (±8.00)
**Peak Vt (l/breath)**	60	2.66 (±0.66)
**Peak Vt/VC (%)**	60	56.98 (±8.67)
**VE/VCO**_**2**_ **-Slope**	60	25.83 (±4.45)

Abbreviations: Bf: Breathing frequency; lbm: Lean Body Mass; VE: Ventilation; VE/VCO_2_-Slope: Ventilation / Volume Carbon dioxide Slope; VO_2_: Volume Oxygen; Vt: Volume Tidal; Vt/VC: Tidal Volume / Vital capacity

The descriptive data tables for PS and SF (S1 and S2 Tables) and for the progression groups (S3 Table) can be found in the appendix. The mean exercising time of the CPET was 09:27 min (±01:50 min). In 80.3% of the conducted CPETs, a RER ≥ 1.15 (Respiratory Exchange Ratio) was achieved (t_0_: 76.6%; t_1_: 85.0%).

### Differences between symptom-free athletes and athletes with persistent symptoms

Table 2 shows the anthropometric data for SF and PS at t0. Age (p = 0.044) and time since infection (p = 0.004) were possible factors influencing the results of the Mann-Whitney-U test. Therefore, age and time since infection were considered as possible confounders in further analyses. The detailed test statistic can be found in the appendix (S4 Table).

**Table 2:** Anthropometric data and test statistic for being a confounder in SF (symptom-free) and PS (persistent symptoms) at t_0_ (first examination date).

Group & Examination Time Point	SF at t_0_	PS at t_0_		
	Mean (±SD)	Mean (±SD)	U	p-value
**Age (years)**	30.06 (±9.21)	37.00 (±12.63)	2.016	**0.044**
**Body Mass (kg)**	73.49 (±11.38)	75.24 (±16.34)	0.117	0.907
**Height (cm)**	177.19 (±8.50)	175.23 (±9.28)	-0.494	0.621
**Body Mass Index (kg/m** ^**2**^ **)**	23.20 (±2.73)	24.33 (±3.87)	0.828	0.408
**Lean Body Mass (kg) (N = 58)**	63.12 (±10.3)	57.58 (±11.94)	-1.583	0.113
**Time since infection (months)**	2.44 (±3.08)	4.73 (±3.96)	2.880	**0.004**

U = test statistic of Mann-Whitney-U-Test, significance level p<0.05.

**Fig 4:**
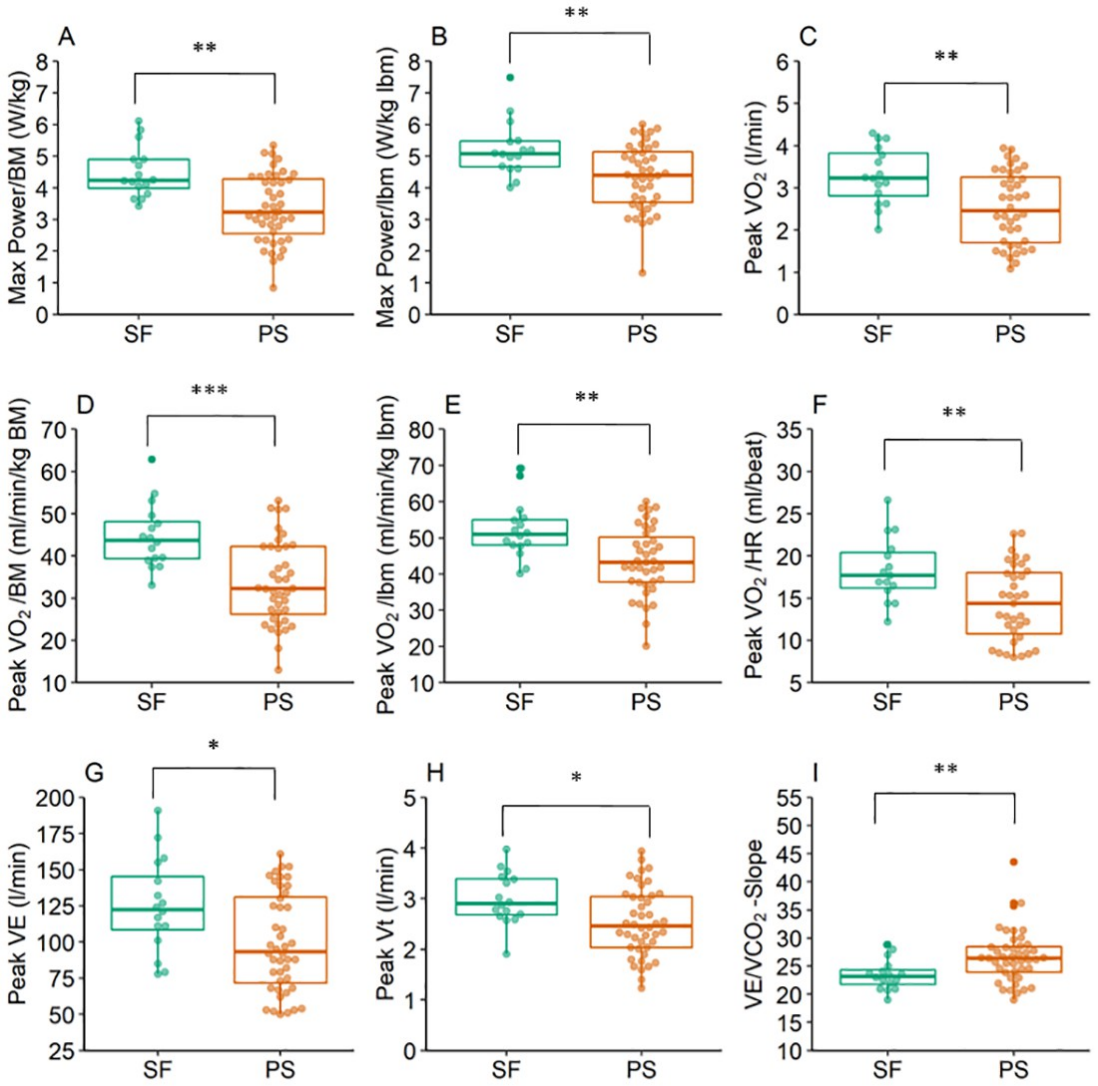
Differences in CPET between SF and PS at study inclusion. SF (symptom-free) had significantly higher mean values for (A) Max Power/BM, (B) Max Power/lbm, (C) Peak VO_2_, Peak VO_2_/BM, (E) Peak VO_2_/lbm, (F) Peak VO_2_/HR, and (G) Peak VE at t_0_ (first examination date). PS (persistent symptoms) had higher mean value for (I) VE/VCO_2_-Slope compared to SF at t_0_. (H) Without confounder, a significantly higher mean value of Peak Vt in SF could be observed. *p<0.05, **p<0.01, ***p<0.001.

Fig 4 shows significant differences between PS and SF at t_0_. Significant differences were found between Max Power/BM (dif = 25.17%, p = 0.001, d = 0.419), Max Power/lbm (dif = 16.86%, p = 0.008, d = 0.347), Peak VO_2_ (dif = 23.17%, p = 0.004, d = 0.385), Peak VO_2_/BM (dif = 24.63%, p<0.001, d = 0.476), Peak VO_2_ /lbm (dif = 16.32%, p = 0.005, d = 0.375) (Peak VO_2_/HR (dif = 21.53%, p = 0.009, d = 0.371), Peak VE (dif = 20.43%, p = 0.021, d = 0.299) and VE/VCO_2_-Slope (dif = -13.77%, p = 0.008, d = 0.340). When considering time since infection as a confounder, a tendency was observed for the variable Peak Vt (p = 0.082). Without consideration of any confounder (dif = 15.67%, p = 0.010, d = 0.331) or with consideration of age (p = 0.012) there was a significant difference, respectively. Even with consideration of time since infection or age, no differences were found for Peak HR, Peak Bf, and Peak Vt/VC.

### Change of CPET parameters over three months per progression group

**Fig 5:**
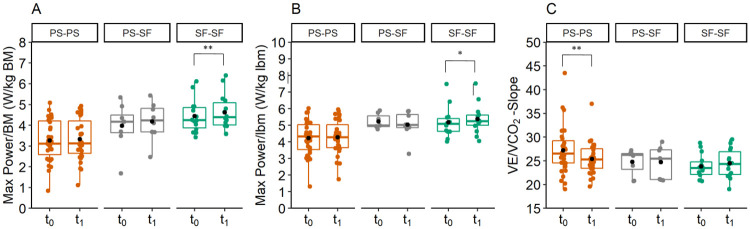
Change of CPET variables over three months. (A) Variable Max Power/BM had in SF-SF (symptom-free–symptom free) significantly higher mean values at t_1_ (three months post first examination) than at t_0_ (first examination date). (B) Variable Max Power/lbm had in SF-SF significantly higher mean values at t_1_ than at t_0_. (C) Variable VE/VCO_2_-Slope had in PS-PS significantly lower mean values at t_1_ than at t_0_. *p<0.05, **p<0.01.

The variable Max Power/BM (Fig 5, Panel A) changed between both examinations in SF-SF (dif = 4.05%, t(13) = -3.239, p = 0.006, d = -0.866). In PS-SF, a tendency (p<0.1) for the variable Max Power/BM could be observed (dif = 4.39%, t(8) = -1.999, p = 0.081, d = -0.666). These athletes were able to generate more power per kg BM at t_1_ than at t_0_.

In SF-SF, a significant difference between the two examination dates was shown for Max Power/lbm (dif = 3.67%, t(13) = -2.847, p = 0.014, d = -0.761) (Fig 5, Panel B). For Max Power/lbm in PS-PS a tendency was observed (dif = 1.94%, t(34) = 1.872, p = 0.061) (Fig 5, Panel B). In PS-SF, this variable did not change. No other variables changed in SF-SF.

In PS-PS a difference for the variable VE/VCO_2_-Slope (dif = -5.98%, t(34) = 2.827, p = 0.008, d = -0.478) was observed. The mean of this variable decreased over time (Fig 5, Panel C). Variable VE/VCO_2_-Slope did not change in SF-SF and PS-SF.

## Discussion

We hypothesized that athletes have a decreased performance after COVID-19 and the performance decrease is related to persistent symptoms. In the follow-up examination, three months later, we observed a decrease in symptoms and a partial recovery of performance.

### Symptoms

Our study showed that at t_0_, 73.3% of athletes who were previously infected with Sars-CoV-2 still suffered from COVID-19-related symptoms, collected with a questionnaire based on the international consensus criteria for myalgic encephalomyelitis / chronic fatigue syndrome. Three categories were most frequently reported: fatigue and performance decrease, neurocognitive disorders and sleeping disorders. Komici et al. [6] conducted a study with competitive athletes in which they found that anosmia was the most common persistent symptom, whereas results similar to ours were shown by Carfi et al. [25]. They observed that 12.6% of the patients who had been hospitalized due to COVID-19 showed ongoing symptoms after 60.3 days since onset of the first COVID-19 symptom. The most reported long-term symptom in their study was fatigue, followed by dyspnea. Goërtz et al. [26] also found persistent symptoms in hospitalized persons and in people with mild or moderate courses. The symptoms most frequently observed by them are equivalent to those in the study by Carfi et al. [25]. Fatigue is also the most reported symptom category in our study, but the second one is sleeping disorders or neurocognitive disorders. These are functional impairments that may get more present over a longer period, for example, sleeping disorders increased in our study over time. A further reason could be the different study population.

Goërtz et al. [26] observed a decline in symptoms for hospitalized and non-hospitalized patients over time. Their cohort was asked about symptoms during the acute infection and about existing symptoms approximately 80 days after the onset of initial symptoms. These patients reported a reduction in fatigue (95% vs. 87%) and dyspnea (90% vs. 71%). This is in line with our results at the follow-up after three months where fewer athletes reported persistent symptoms. In addition, in our study, the number of persistent symptoms per person also declined in most of the cases. However, the symptoms in the categories of sleeping disorders, psychological-related items and immunological disorders increased. The origins of mental illness after a Sars-CoV-2 infection can be manifold, e.g. neurotrophic factors or impaired learning and memory [27]. Raveendran et al. [28] showed that psychological-related items are common for Long-COVID, which is in accordance with our results. This could be indicative of a development of psychological items due to an ongoing inflammation in the brain or the persistent low physical capability, which can negatively affect mental health [29]. A recent review by Haller et al. [30] showed that persistent fatigue is a risk factor for a decreased life quality and work capacity. Furthermore, they observed that pre-existing psychological disorders also increase the risk for the Post-COVID syndrome [30]. Therefore, our results that psychological disorders increased over time, could indicate that the life quality decreases with persistent fatigue. Therefore, it seems necessary to monitor not only the performance capability but also the mental well-being, for example with the EQ-5D questionnaire [31], and for symptom collection, the International consensus CFS questionnaire could be used [24].

### Prediction of peak oxygen consumption before infection

The results of the comparison between predVO_2_ and the measured VO_2_/BM show that COVID-19 leads to a decrease in performance, regardless of whether or not persistent symptoms exist. However, the performance decline is smaller in symptom-free athletes than in athletes with persistent symptoms. Furthermore, athletes with a higher estimated predVO_2_ are less likely to suffer from persistent symptoms. This is consistent with the findings by Massey et al. [32] who also found a lower prevalence of Long-Covid in athletes compared with the general population.

### Performance in CPET

19.7% of our conducted CPETs ended without objective total exhaustion (RER<1.15), although all of our athletes reported subjective exhaustion at the end of the test. This suggests that some individuals were unable to exhaust themselves due to unknown mechanisms.

Additionally, we found decreased Max Power/lbm, Peak VO_2_/lbm, Peak VO_2_, and Peak VO_2_/HR, in athletes with symptoms compared to athletes without symptoms. This declined performance capability stands in contrast to the results by Anastasio et al. [33], who could not find differences in oxygen consumption at the maximum load between elite cross-country athletes with a mild-moderate disease course and healthy peers. However, they found differences at the ventilatory threshold 1 (VT1), which is an indicator of the aerobic capacity and thus it has an impact on the general performance capability. This result is in line with the result of a reduced Peak VO_2_/HR in our study because a reduced Peak VO_2_/HR can be, among others, an indicator of a limited aerobic capacity. A difference to our study is the shorter average period between infection and examination, the training status (all of their study participants competed in national and international competitions) as well as the gender distribution (77% male). As mentioned above, they only included mild-moderate COVID-19 courses and thus they excluded all patients who suffered from dyspnea. This could explain why no differences were found for other ventilatory variables.

Our results are in accordance with the results by Skjørten et al. [34]. They also observed decreased performance capability parameters, like peak oxygen uptake and oxygen pulse, three months after the Sars-CoV-2 infection in hospitalized patients. In addition, reduced Peak VO_2_ was observed by Debeaumont et al. [22] in a CPET six months after infection, while Barbagelata et al. [35] focused on patients with and without Post-COVID-19 syndrome and also found a lower Peak VO_2_ in patients with Post-COVID-19 syndrome. This result is in accordance with our result with an above-average fit cohort: Athletes with persistent symptoms have a lower Peak VO_2_/BM than athletes who are symptom-free. In contrast to this, Komici et al. [6] could not find any significant differences in CPET between competitive athletes who were either post-acute Sars-CoV-2 infection or who had not been infected. However, they did not cluster in accordance to their persistent symptoms.

### Ventilatory parameters of CPET

In our study, the ventilatory parameters Peak VE, Peak Vt and VE/VCO_2_-Slope differed significantly between PS and SF at t_0_. It was already shown that besides a reduced VO_2_ peak consumption, patients with persistent dyspnea have a higher VE/VCO_2_-Slope and a higher PET_CO2_ than symptom-free patients [14]. While Ladlow et al. [36] did not find an association between reported symptoms by the patients and the perceived functional limitation and dysautonomia, they found an association between dysautonomia and reduced work rate, VO_2_ peak and VE/VCO_2_-Slope. Further studies also showed an increased VE/VCO_2_-Slope [37, 38]. A high VE/VCO_2_-Slope could be a result of hyperventilation, a lung obstruction or reduced lung perfusion [39]. However, the breathing frequency did not differ significantly between PS and SF, but Peak Vt was lower in PS compared to SF. This could indicate that these athletes are not able to inhale the same volume compared to athletes from SF. Decreased Peak Vt can be caused by prolonged sedentary behavior due to persistent symptoms which limit activity during daily life. Due to this circumstance, the auxiliary respiratory muscles can degenerate and this could result in a less efficient and less powerful breathing technique [39]. However, the ratio Peak Vt/VC was not significantly different between both groups. Therefore, further research regarding a potential auxiliary respiratory muscle degeneration may be useful.

### CPET variables of the three months follow-up

To be able to provide information on how performance develops over time, long-term monitoring is necessary. Recent published studies and recommendations also state the need for long time monitoring [21, 40, 41]. Studies with long-term monitoring and repeated examinations of patients and athletes are rare. In our monitoring study, we showed that there is an improvement of the VE/VCO_2_-Slope and a tendency of improvement for Max Power/lbm for athletes with persistent symptoms (PS-PS). A tendency of enhancement of Max Power/BM was shown in PS-SF, and in SF-SF an improvement of Max Power/BM and Max Power/lbm. The results for SF-SF could indicate that a larger amount of training, after isolation and protection of the body due to the infection, led to an improvement of the performance over time that had been decreased due to acute illness. It can be assumed that athletes are more likely to return to sport after an acute infection than people with a more sedentary lifestyle.

The observed decrease of VE/VCO_2_-Slope in PS-PS indicates that there is a slow regeneration of the ventilatory efficiency, despite persistent symptoms. It is possible that there are still persistent symptoms while the intensity of the symptoms declines. Although we did not assess the intensity, this would be in accordance with the results by Rooney et al. [42], who showed that there is an ongoing but still incomplete regeneration occurring in a proportion of previously infected individuals. A change in the VE/VCO_2_-Slope can result from a change in ventilation. However, we did not see any significant differences in Peak VE, Peak Vt, or Peak Bf. Therefore, we assume that the improved VE/VCO_2_-Slope is caused by a better lung perfusion. Furthermore, the tendency of improvement in Max Power/lbm and the descriptive data showed an improvement in the general performance capacity (Peak VO_2_, Peak VO_2_/BM, VO_2_/HR), which may indicate a general recovery.

In PS-SF, a tendency of improvement for Max Power/BM was found. This is in accordance with the results of PS-PS. The performance capacity increased slightly over time. Furthermore, descriptive data showed slightly improved ventilatory parameters. Regarding the ventilatory parameters, it is possible that the athletes declared being symptom-free at t_1_ because they potentially felt better due to less restriction during ventilation.

## Limitations

Nine of 60 athletes in the study met the criteria of the PS-SF group. This relatively low number is a probable reason why no significant results could be shown here. However, a tendency could be shown that might indicate that significant differences could be shown with a higher number of athletes. One further limitation was that the intensity of the symptoms was not asked. Furthermore, the partly unknown medical history and the athletes’ unknown behavior between the examinations reduce the explanatory power of this study’s results. However, the different training behavior between the two examination dates is difficult to control due to the different duration of symptoms. Although the participants were selected according to the inclusion criteria, a certain heterogeneity of the group could not be avoided.

## Conclusion

The study showed that after COVID-19, 70.3% of athletes stated having symptoms in the questionnaire at the first examination and 61.7% had persistent symptoms at the second examination. In both groups, the maximal oxygen uptake was decreased compared to predicted maximal oxygen uptake before infection. Moreover, the reduction in VO_2_/BM in symptom-free athletes was smaller than in athletes with persistent symptoms. Athletes with a higher maximal oxygen uptake before infection were less likely to report persistent symptoms after a Sars-CoV-2 infection.

The study showed differences in ventilatory (VE, VE/VCO_2_-Slope) as well as in general performance parameters (e.g. Max Power/BM, PeakVO_2_/BM and Peak HR/VO_2_) between symptom-free athletes and athletes with persistent symptoms. Which area (respiratory, cardiac and/or muscular) is restricted and how long the restrictions last seems to be individual. Nevertheless, the decrease of the respiratory equivalent for athletes with long-term symptoms indicate a slow recovery of the respiratory tract. To explain the mechanism of this fact further studies are needed. In further studies, possible correlations of symptom categories, and CPET parameters, for example, Max Power/lbm, Peak VO_2_/kg or VE/VCO_2_-Slope should be investigated. Furthermore, there is a need to investigate the reason for performance decline and long-lasting symptoms and the reasons why a number of people suffer from persistent symptoms for such a long period and other do not. To gain further insights into athletes’ recovery and the progression of persistent symptoms, a longer period of monitoring as well as a still higher number of patients are needed.

## Supporting information

S1 Table Descriptive data of the CPET variables for SF (symptom-free) and PS (persistent symptoms) at t_0_ (first examination date). Abbreviations: Bf: Breathing frequency; lbm: Lean Body Mass; VE: Ventilation; VE/VCO2-Slope: Ventilation / Volume Carbon dioxide Slope; VO2: Volume Oxygen; Vt: Volume Tidal; Vt/VC: Tidal Volume / Vital capacity.
(DOCX)

S2 Table Descriptive data of the CPET variables for SF (symptom-free) and PS (persistent symptoms) at t_1_ (three months post first examination). Abbreviations: Bf: Breathing frequency; lbm: Lean Body Mass; VE: Ventilation; VE/VCO2-Slope: Ventilation / Volume Carbon dioxide Slope; VO2: Volume Oxygen; Vt: Volume Tidal; Vt/VC: Tidal Volume / Vital capacity.
(DOCX)

S3 Table Descriptive data of the CPET variables for SF-SF (symptom-free—symptom free), PS-PS (persistent symptoms—persistent symptoms) and PS-SF (persistent symptoms–symptom-free) at t_0_ (first examination date) and t_1_ (three months post first examination). Abbreviations: Bf: Breathing frequency; lbm: Lean Body Mass; VE: Ventilation; VE/VCO2-Slope: Ventilation / Volume Carbon dioxide Slope; VO2: Volume Oxygen; Vt: Volume Tidal; Vt/VC: Tidal Volume / Vital capacity.
(DOCX)

S4 Table Test statistic for the Mann-Whitney-U test without confounder, considering time since infection or considering age. *for each test: df = 1Abbreviations: β: beta weight; Bf: Breathing frequency; lbm: Lean Body Mass; tsi: time since infection; VE: Ventilation; VE/VCO2-Slope: Ventilation / Volume Carbon dioxide Slope; VO2: Volume Oxygen; Vt: Volume Tidal; Vt/VC: Tidal Volume / Vital capacity.
(DOCX)

## Abbreviations

Bf: Breathing Frequency
BM: Body Mass
CFS: Chronic Fatigue Syndrome
CoSmo-S: COVID-19 in elite sports – A multicenter cohort study
COVID-19: Corona Virus Disease 2019
CPET: Cardiopulmonary Exercise Test
IC: Inspiratory Capacity
lbm: Lean Body Mass
MVV: Maximal Voluntary Volume
PETCO_2_: Partial End tidal Carbon dioxide
predVO_2_: Prediction of Volume Oxygen before infection
PS: Persistent Symptoms Group
RER: Respiratory Exchange Ratio
SF: Symptom-free Group
t_0_: Study inclusion, first examination
t_1_: Three months post t_0_, second examination
VE/VCO_2_-Slope: Ventilation / Volume Carbon dioxide Slope
VE: Ventilation
VO_2_: Volume Oxygen
Vt/VC: Tidal Volume / Vital capacity
Vt: Volume Tidal
VT1: Ventilatory Threshold 1
