# Supplementary material for: Recovery of performance and persistent symptoms in athletes after COVID-19
Source: PLoS One. 2022 Dec 7;17(12):e0277984. doi: 10.1371/journal.pone.0277984 (PMC9728914; doi:10.1371/journal.pone.0277984)
Supplement: S1 Table — Abbreviations: Bf: Breathing frequency; lbm: Lean Body Mass; VE: Ventilation; VE/VCO2-Slope: Ventilation / Volume Carbon dioxide Slope; VO2: Volume Oxygen; Vt: Volume Tidal; Vt/VC: Tidal Volume / Vital capacity. (DOCX) [file pone.0277984.s001.docx]

**S1 Table. Descriptive data of the CPET variables for SF (symptom-free) and PS (persistent symptoms) at t_0_ (first examination date).**

|  | **t_0_** | | | | | | | |
| --- | --- | --- | --- | --- | --- | --- | --- | --- |
|  | **N** | | **Minimum** | | **Maximum** | | **Mean (±SD)** | |
| **Group** | **SF** | **PS** | **SF** | **PS** | **SF** | **PS** | **SF** | **PS** |
| **Max Power/BM (W/kg BM)** | 16 | 44 | 3.42 | 0.84 | 6.12 | 5.34 | 4.49  (±0.80) | 3.36  (±1.07) |
| **Max Power/lbm (W/kg lbm)** | 16 | 42 | 4.02 | 1.31 | 7.48 | 6.01 | 5.22  (±0.86) | 4.34  (±1.04) |
| **Peak VO_2_ (l/min)** | 16 | 40 | 2.01 | 1.09 | 4.30 | 3.94 | 3.28  (±0.68) | 2.52  (±0.85) |
| **Peak VO_2_/BM (ml/min/kg BM)** | 16 | 40 | 33.1 | 13.00 | 62.9 | 53.10 | 44.66  (±7.65) | 33.66  (±9.92) |
| **Peak VO_2_ /lbm (ml/min/ kg lbm)** | 16 | 39 | 40.20 | 20.15 | 69.15 | 60.06 | 52.07  (±7.85) | 43.57  (±9.48) |
| **Peak HR**  **(1/min)** | 15 | 37 | 157.00 | 111.00 | 197.00 | 195.00 | 175.87  (±10.92) | 170.00  (±16.02) |
| **Peak VO_2_/HR (ml/beat)** | 15 | 35 | 12.20 | 8.00 | 26.60 | 22.70 | 18.35  (±3.82) | 14.40  (±4.50) |
| **Peak VE**  **(l/min)** | 16 | 44 | 78.00 | 50.00 | 191.00 | 161.00 | 125.25  (±32.53) | 99.66  (±33.89) |
| **Peak Bf**  **(1/min)** | 16 | 44 | 24.00 | 23.00 | 61.00 | 52.00 | 41.63  (±9.89) | 39.09  (±7.21) |
| **Peak Vt**  **(l/breath)** | 16 | 44 | 1.91 | 1.23 | 3.97 | 3.94 | 3.00  (±0.51) | 2.53  (±0.67) |
| **Peak Vt/VC**  **(%)** | 16 | 44 | 45.00 | 37.00 | 67.00 | 97.00 | 56.12  (±6.59) | 57.30  (±9.36) |
| **VE/VCO_2_-Slope** | 16 | 44 | 19.00 | 19.00 | 28.80 | 43.50 | 23.46  (±2.70) | 26.69  (±4.66) |

Abbreviations: Bf: Breathing frequency; lbm: Lean Body Mass; VE: Ventilation; VE/VCO_2_-Slope: Ventilation / Volume Carbon dioxide Slope; VO_2_: Volume Oxygen; Vt: Volume Tidal; Vt/VC: Tidal Volume / Vital capacity
