# Supplementary material for: Recovery of performance and persistent symptoms in athletes after COVID-19
Source: PLoS One. 2022 Dec 7;17(12):e0277984. doi: 10.1371/journal.pone.0277984 (PMC9728914; doi:10.1371/journal.pone.0277984)
Supplement: S2 Table — Abbreviations: Bf: Breathing frequency; lbm: Lean Body Mass; VE: Ventilation; VE/VCO2-Slope: Ventilation / Volume Carbon dioxide Slope; VO2: Volume Oxygen; Vt: Volume Tidal; Vt/VC: Tidal Volume / Vital capacity. (DOCX) [file pone.0277984.s002.docx]

**S2 Table. Descriptive data of the CPET variables for SF (symptom-free) and PS (persistent symptoms) at t_1_ (three months post first examination).**

|  | **t_1_** | | | | | | | |
| --- | --- | --- | --- | --- | --- | --- | --- | --- |
|  | **N** | | **Minimum** | | **Maximum** | | **Mean (±SD)** | |
| **Group** | **SF** | **PS** | **SF** | **PS** | **SF** | **PS** | **SF** | **PS** |
| **Max Power/BM (W/kg BM)** | 23 | 37 | 2.46 | 1.11 | 6.40 | 6.58 | 4.49 (±0.88) | 3.37 (±1.09) |
| **Max Power/lbm (W/kg lbm)** | 23 | 37 | 3.28 | 1.74 | 7.52 | 7.27 | 5.25  (±0.84) | 4.32  (±1.09) |
| **Peak VO_2_ (l/min)** | 23 | 35 | 2.22 | 1.03 | 4.47 | 4.46 | 3.26  (±0.65) | 2.49  (±0.88) |
| **Peak VO_2_/BM (ml/min/kg BM)** | 23 | 35 | 28.90 | 13.90 | 60.10 | 65.65 | 43.82 (±8.45) | 33.84 (±10.43) |
| **Peak VO_2_ /lbm (ml/min/ kg lbm)** | 23 | 35 | 38.15 | 21.74 | 70.49 | 72.05 | 51.38  (±7.76) | 43.28  (±10.39) |
| **Peak HR**  **(1/min)** | 22 | 36 | 160.00 | 125.00 | 190.00 | 195.00 | 176.82  (±8.96) | 168.47  (±16.101) |
| **Peak VO_2_/HR (ml/beat)** | 22 | 37 | 12.10 | 6.70 | 26.60 | 24.60 | 18.57  (±4.02) | 14.58  (±4.76) |
| **Peak VE**  **(l/min)** | 23 | 37 | 73.00 | 47.00 | 195.00 | 183.00 | 127.00  (±27.49) | 100.19  (±37.60) |
| **Peak Bf**  **(1/min)** | 23 | 37 | 26.00 | 25.00 | 61.00 | 63.00 | 43.13  (±9.29) | 39.81  (±9.44) |
| **Peak Vt**  **(l/breath)** | 23 | 37 | 1.96 | 1.09 | 4.23 | 4.03 | 2.97  (±0.54) | 2.50  (±0.75) |
| **Peak Vt/VC**  **(%)** | 21 | 36 | 47.00 | 35.00 | 71.00 | 76.00 | 55.86  (±5.77) | 56.61  (±9.28) |
| **VE/VCO_2_-Slope** | 23 | 37 | 19.00 | 19.60 | 29.50 | 37.00 | 24.37  (±3.14) | 25.45  (±3.70) |

Abbreviations: Bf: Breathing frequency; lbm: Lean Body Mass; VE: Ventilation; VE/VCO_2_-Slope: Ventilation / Volume Carbon dioxide Slope; VO_2_: Volume Oxygen; Vt: Volume Tidal; Vt/VC: Tidal Volume / Vital capacity
