# Supplementary material for: Recovery of performance and persistent symptoms in athletes after COVID-19
Source: PLoS One. 2022 Dec 7;17(12):e0277984. doi: 10.1371/journal.pone.0277984 (PMC9728914; doi:10.1371/journal.pone.0277984)
Supplement: S3 Table — Abbreviations: Bf: Breathing frequency; lbm: Lean Body Mass; VE: Ventilation; VE/VCO2-Slope: Ventilation / Volume Carbon dioxide Slope; VO2: Volume Oxygen; Vt: Volume Tidal; Vt/VC: Tidal Volume / Vital capacity. (DOCX) [file pone.0277984.s003.docx]

**S3 Table. Descriptive data of the CPET variables for SF-SF (symptom-free - symptom free), PS-PS (persistent symptoms - persistent symptoms) and PS-SF (persistent symptoms – symptom-free) at t_0_ (first examination date) and t_1_ (three months post first examination).**

|  | **SF-SF** | | | | **PS-PS** | | | | **PS-SF** | | | |
| --- | --- | --- | --- | --- | --- | --- | --- | --- | --- | --- | --- | --- |
|  | **N** | | **Mean (SD)** | | **N** | | **Mean (SD)** | | **N** | | **Mean (SD)** | |
|  | **t_0_** | **t_1_** | **t_0_** | **t_1_** | **t_0_** | **t_1_** | **t_0_** | **t_1_** | **t_0_** | **t_1_** | **t_0_** | **t_1_** |
| **Max Power/BM (W/kg BM)** | 14 | 14 | 4.44 (±0.80) | 4.62 (±0.87) | 35 | 35 | 3.17 (±0.99) | 3.26 (±0.96) | 9 | 9 | 4.10 (±1.10) | 4.28 (±0.92) |
| **Max Power/lbm (W/kg lbm)** | 14 | 14 | 5.18  (±0.86) | 5.37  (±0.85) | 34 | 35 | 4.13  (±1.02) | 4.21  (±0.99) | 8 | 9 | 5.27  (±0.43) | 5.08  (±0.79) |
| **Peak VO_2_ (l/min)** | 14 | 14 | 3.29  (±0.69) | 3.34  (±0.67) | 32 | 33 | 2.35  (±0.83) | 2.41  (±0.83) | 8 | 9 | 3.18  (±0.66) | 3.13  (±0.57) |
| **Peak VO_2_/BM (ml/min/kg BM)** | 14 | 14 | 44.42 (±7.74) | 45.45 (±8.10) | 32 | 33 | 31.24 (±8.87) | 32.65 (±8.99) | 8 | 9 | 43.35 (±8.13) | 41.29 (±8.82) |
| **Peak VO_2_ /lbm (ml/min/ kg lbm)** | 14 | 14 | 51.95  (±7.91) | 52.93  (±7.66) | 31 | 33 | 41.45  (±9.10) | 42.21  (±9.30) | 8 | 9 | 51.78  (±5.98) | 48.97  (±7.23) |
| **Peak HR**  **(1/min)** | 13 | 14 | 175.62  (±11.23) | 175.50  (±8.96) | 31 | 34 | 168.13  (±16.59) | 167.91  (±16.16) | 6 | 8 | 179.67  (±7.99) | 179.13  (±8.41) |
| **Peak VO_2_/HR (ml/beat)** | 13 | 14 | 18.36  (±3.90) | 19.09  (±3.97) | 29 | 35 | 13.69  (±4.22) | 14.21  (±4.58) | 6 | 8 | 17.80  (±4.61) | 17.66  (±4.00) |
| **Peak VE**  **(l/min)** | 14 | 14 | 126.21  (±30.05) | 127.86  (±28.98) | 35 | 35 | 94.80  (±33.72) | 97.17  (±35.65) | 9 | 9 | 118.56  (±28.92) | 125.67  (±24.61) |
| **Peak Bf**  **(1/min)** | 14 | 14 | 41.50  (±7.46) | 42.29  (±9.28) | 35 | 35 | 38.46  (±7.00) | 39.34  (±8.78) | 9 | 9 | 41.56  (±7.91) | 44.44  (±9.14) |
| **Peak Vt**  **(l/breath)** | 14 | 14 | 3.01  (±0.51) | 3.05  (±0.56) | 35 | 35 | 2.45  (±0.70) | 2.46  (±0.74) | 9 | 9 | 2.82  (±0.42) | 2.84  (±0.47) |
| **Peak Vt/VC**  **(%)** | 14 | 13 | 56.43  (±5.85) | 54.92  (±5.11) | 35 | 34 | 56.91  (±10.12) | 56.44  (±9.17) | 9 | 8 | 58.78  (±5.04) | 57.38  (±6.52) |
| **VE/VCO_2_-Slope** | 14 | 14 | 23.84  (±2.48) | 24.47  (±2.96) | 35 | 35 | 27.26  (±4.92) | 25.63  (±3.72) | 9 | 9 | 24.48  (±2.68) | 24.21  (±3.42) |

Abbreviations**:** Bf: Breathing frequency; lbm: Lean Body Mass; VE: Ventilation; VE/VCO_2_-Slope: Ventilation / Volume Carbon dioxide Slope; VO_2_: Volume Oxygen; Vt: Volume Tidal; Vt/VC: Tidal Volume / Vital capacity
