# Supplementary material for: Recovery of performance and persistent symptoms in athletes after COVID-19
Source: PLoS One. 2022 Dec 7;17(12):e0277984. doi: 10.1371/journal.pone.0277984 (PMC9728914; doi:10.1371/journal.pone.0277984)
Supplement: S4 Table — *for each test: df = 1Abbreviations: β: beta weight; Bf: Breathing frequency; lbm: Lean Body Mass; tsi: time since infection; VE: Ventilation; VE/VCO2-Slope: Ventilation / Volume Carbon dioxide Slope; VO2: Volume Oxygen; Vt: Volume Tidal; Vt/VC: Tidal Volume / Vital capacity. (DOCX) [file pone.0277984.s004.docx]

**S4 Table. Test statistic for the Mann-Whitney-U test without confounder, considering time since infection or considering age.**

| **t_0_** | | | | | | | | | | |
| --- | --- | --- | --- | --- | --- | --- | --- | --- | --- | --- |
|  | **Without confounder** | | **Confounder: Time since infection** | | | | **Confounder: Age** | | | |
|  | **U** | **p-value** | **β***  **(tsi)** | **p-value**  **(tsi)** | **β***  **(Group)** | **p-value**  **(Group)** | **β***  **(Age)** | **p-value**  **(Age)** | **β***  **(Group)** | **p-value**  **(Group)** |
| **Max Power/BM (W/kg BM)** | -3.243 | **0.001** | -1.784 | 0.080 | 3.647 | **<0.001** | -2.230 | 0.030 | 3.574 | **<0.001** |
| **Max Power/lbm (W/kg lbm)** | -2.644 | **0.008** | -1.407 | 0.165 | 2.779 | **0.007** | -1.500 | 0.139 | 2.677 | **0.010** |
| **Peak VO_2_ (l/min)** | -2.844 | **0.004** | -0.221 | 0.826 | 2.674 | **0.010** | -1.781 | 0.081 | 2.826 | **0.007** |
| **Peak VO_2_/BM (ml/min/kg BM)** | -3.564 | **<0.001** | -1.644 | 0.106 | 3.634 | **<0.001** | -2.430 | 0.019 | 3.508 | **<0.001** |
| **Peak VO_2_ /lbm (ml/min/ kg lbm)** | -2.780 | **0.005** | -0.869 | 0.389 | 2.986 | **0.004** | -2.341 | 0.023 | 2.599 | **0.012** |
| **Peak HR**  **(1/min)** | -0.950 | 0.342 | -0.307 | 0.760 | 1.163 | 0.251 | -3.284 | 0.002 | 0.806 | 0.424 |
| **Peak VO_2_/HR (ml/beat)** | -2.626 | **0.009** | -0.439 | 0.663 | 2.529 | **0.015** | -1-756 | 0.086 | 2.503 | **0.016** |
| **Peak VE**  **(l/min)** | -2.316 | **0.021** | -0.904 | 0.370 | 2.025 | **0.048** | -1.148 | 0.256 | 2.201 | **0.032** |
| **Peak Bf**  **(1/min)** | -0.762 | 0.446 | -0.288 | 0.775 | 0.871 | 0.387 | -1.329 | 0.189 | 0.616 | 0.541 |
| **Peak Vt**  **(l/breath)** | -2.566 | **0.010** | -0.802 | 0.426 | 1.770 | 0.082 | -0.359 | 0.721 | 2.598 | **0.012** |
| **Peak Vt/VC**  **(%)** | 0.335 | 0.738 | 0.229 | 0.819 | -0.208 | 0.836 | 1.884 | 0.065 | 0.250 | 0.803 |
| **VE/VCO_2_-Slope** | 2.633 | **0.008** | 0.304 | 0.762 | -3.104 | **0.003** | 1.057 | 0.295 | -2.776 | **0.007** |

*for each test: df=1

Abbreviations: β: beta weight; Bf: Breathing frequency; lbm: Lean Body Mass; tsi: time since infection; VE: Ventilation; VE/VCO_2_-Slope: Ventilation / Volume Carbon dioxide Slope; VO_2_: Volume Oxygen; Vt: Volume Tidal; Vt/VC: Tidal Volume / Vital capacity
